# (*E*)-1-{4-[Bis(4-meth­oxy­phen­yl)meth­yl]piperazin-1-yl}-3-(4-methyl­phen­yl)prop-2-en-1-one

**DOI:** 10.1107/S1600536811039353

**Published:** 2011-09-30

**Authors:** Yan Zhong, Bin Wu

**Affiliations:** a School of Chemistry and Chemical Engineering, Southeast University, Sipailou No.2 Nanjing, Nanjing 210096, People’s Republic of China; b School of Pharmacy, Nanjing Medical University, Hanzhong Road No.140 Nanjing, Nanjing 210029, People’s Republic of China

## Abstract

In the title mol­ecule, C_29_H_32_N_2_O_3_, the piperazine ring has a chair conformation. The amide N atom is almost planar (bond angle sum = 359.5°), whereas the other N atom is clearly pyramidal (bond angle sum = 330.4°). The dihedral angle between the meth­oxy­benzene rings is 81.29 (16)°. In the crystal, mol­ecules are linked by C—H⋯O hydrogen bonds.

## Related literature

For structures and properties of cinnamic acid derivatives, see: Shi *et al.* (2005 ▶); Qian *et al.* (2010 ▶). For the synthesis, see: Wu *et al.* (2008 ▶). For related structures, see: Mouillé *et al.* (1975) ▶; Teng *et al.* (2011 ▶).
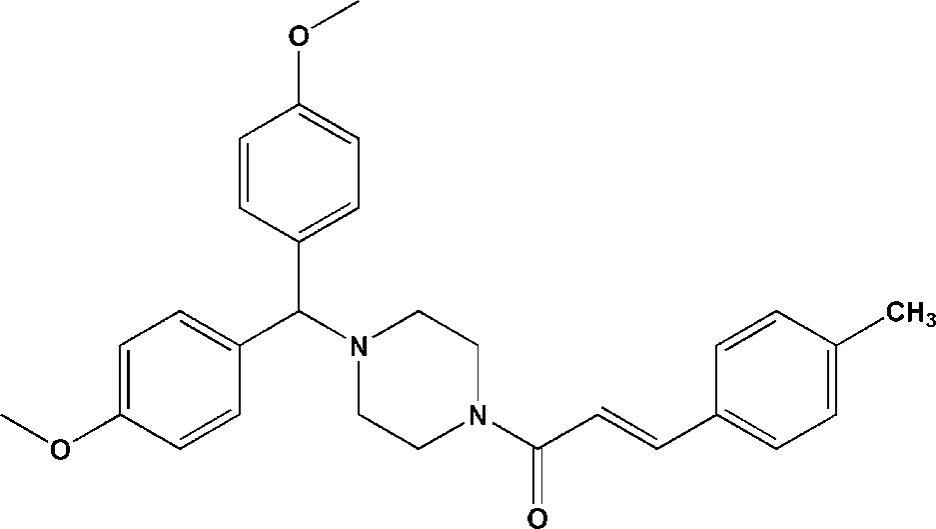


## Experimental

### 

#### Crystal data

C_29_H_32_N_2_O_3_
*M* *_r_* = 456.57
Monoclinic, 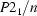

*a* = 10.114 (2) Å
*b* = 11.867 (2) Å
*c* = 21.573 (4) Å
β = 97.12 (3)°
*V* = 2569.3 (9) Å^3^
*Z* = 4
Mo *K*α radiation
μ = 0.08 mm^−1^
*T* = 293 K
0.30 × 0.20 × 0.20 mm

#### Data collection

Enraf–Nonius CAD-4 diffractometer
5002 measured reflections
4718 independent reflections
2269 reflections with *I* > 2σ(*I*)
*R*_int_ = 0.037
3 standard reflections every 200 reflections  intensity decay: 1%

#### Refinement

*R*[*F* ^2^ > 2σ(*F* ^2^)] = 0.061
*wR*(*F* ^2^) = 0.189
*S* = 1.01
4718 reflections
308 parameters
H-atom parameters constrained
Δρ_max_ = 0.17 e Å^−3^
Δρ_min_ = −0.16 e Å^−3^

### 

Data collection: *CAD-4 EXPRESS* (Enraf–Nonius, 1989 ▶); cell refinement: *CAD-4 EXPRESS*; data reduction: *XCAD4* (Harms & Wocadlo, 1995 ▶); program(s) used to solve structure: *SHELXS97* (Sheldrick, 2008 ▶); program(s) used to refine structure: *SHELXL97* (Sheldrick, 2008 ▶); molecular graphics: *SHELXTL* (Sheldrick, 2008 ▶); software used to prepare material for publication: *SHELXL97*.

## Supplementary Material

Crystal structure: contains datablock(s) I, global. DOI: 10.1107/S1600536811039353/hb6419sup1.cif

Structure factors: contains datablock(s) I. DOI: 10.1107/S1600536811039353/hb6419Isup2.hkl

Supplementary material file. DOI: 10.1107/S1600536811039353/hb6419Isup3.cml

Additional supplementary materials:  crystallographic information; 3D view; checkCIF report

## Figures and Tables

**Table 1:** Hydrogen-bond geometry (Å, °)

*D*—H⋯*A*	*D*—H	H⋯*A*	*D*⋯*A*	*D*—H⋯*A*
C11—H11*A*⋯O1^i^	0.93	2.60	3.360 (4)	140
C22—H22*A*⋯O3^ii^	0.93	2.59	3.483 (4)	160

Symmetry codes: (i) 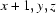; (ii) 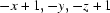.

**Fig. 1.**
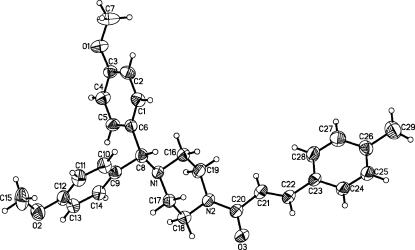
The molecular structure of the title compound. Displacement ellipsoids for non-H atoms are drawn at the 70% probability level.

**Fig. 2.**
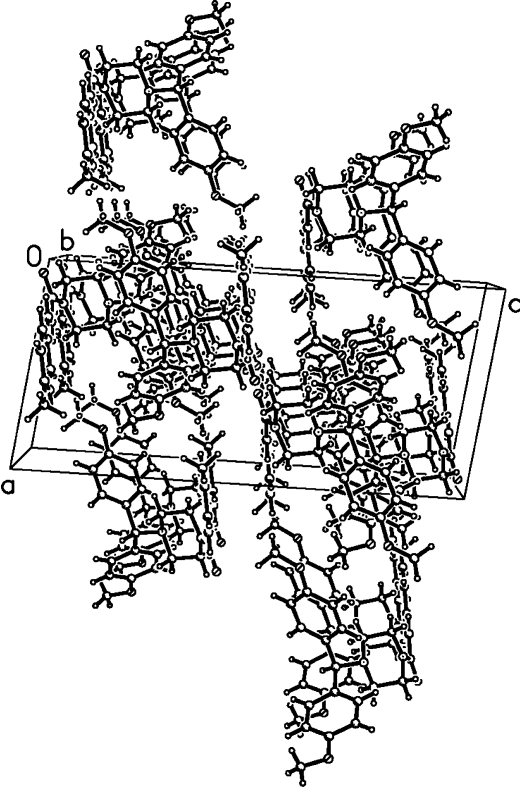
Packing diagram of the title compound.

## supplementary crystallographic information

### Comment

Recently, many compounds containing a cinnamoyl moiety have drawn much attention owing to their significant pharacological properties such as antimicrobial, anticancer and neuroprotective activities (Shi *et al.*, 2005; Qian *et al.*, 2010). As a part of our ongoing study of the substituent effect on the stuctures of cinnamide derivatives, we report herein the crystal structure of the title compound. The molecule of the title compound exists an E configulation with respect to the C21=C22 ethene bond [1.318 (4)] and the torsion angle C20—C21—C22—C23 = -178.7 (3). The piperazine ring adopts a chair conformation. In the crystal, moleculaes are linked by intermolecular C—H···O interactions.

### Experimental

The synthesis follows the method of Wu *et al.* (2008). The title compound was prepared by stirring a mixture of (*E*)-3-(4-methylphenyl)acrylic acid (0.649 g; 4 mmol), dimethyl sulfoxide (2 ml) and dichloromethane (30 ml) for 6 h at room temperature. The solvent was removed under reduced pressure. The residue was dissolved in acetone (15 ml) and reacted with 1-(bis(4-methoxyphenyl)methyl) piperazine (1.874 g; 6 mmol) in the presence of triethylamine (5 ml) for 12 h at room temperature. The resultant mixture was cooled. The solid, (*E*)-1-(4-(bis(4-methoxyphenyl)methyl) piperazin-1-yl)-3- (4-methylphenyl)prop-2-en-1-one obtained was filtered and was recrystallized from ethanol. The colorless single crystals of the title compound used in *x*-ray diffraction studies were grown in ethanol by a slow evaporation at room temperature.

### Refinement

All non-hydrogen atoms were refined anisotropically. All hydrogen atoms were positioned geometrically with C—H distances ranging from 0.93 Å to 0.98 Å and refined as riding on their parent atoms with *U*ĩso~(H) = 1.2 or 1.5*U*~eq~ of the carrier atom.

### Crystal data

**Table:**

C_29_H_32_N_2_O_3_	*F*(000) = 976
*M**_r_* = 456.57	*D*_x_ = 1.180 Mg m^−^^3^
Monoclinic, *P*2_1_/*n*	Mo *K*α radiation, λ = 0.71073 Å
Hall symbol: -P 2yn	Cell parameters from 25 reflections
*a* = 10.114 (2) Å	θ = 9–13°
*b* = 11.867 (2) Å	µ = 0.08 mm^−^^1^
*c* = 21.573 (4) Å	*T* = 293 K
β = 97.12 (3)°	Block, colorless
*V* = 2569.3 (9) Å^3^	0.30 × 0.20 × 0.20 mm
*Z* = 4	

### Data collection

**Table:**

Enraf–Nonius CAD-4 diffractometer	*R*_int_ = 0.037
Radiation source: fine-focus sealed tube	θ_max_ = 25.4°, θ_min_ = 1.9°
graphite	*h* = 0→12
ω/2θ scans	*k* = 0→14
5002 measured reflections	*l* = −25→25
4718 independent reflections	3 standard reflections every 200 reflections
2269 reflections with *I* > 2σ(*I*)	intensity decay: 1%

### Refinement

**Table:**

Refinement on *F*^2^	Secondary atom site location: difference Fourier map
Least-squares matrix: full	Hydrogen site location: inferred from neighbouring sites
*R*[*F*^2^ > 2σ(*F*^2^)] = 0.061	H-atom parameters constrained
*wR*(*F*^2^) = 0.189	*w* = 1/[σ^2^(*F*_o_^2^) + (0.090*P*)^2^] where *P* = (*F*_o_^2^ + 2*F*_c_^2^)/3
*S* = 1.01	(Δ/σ)_max_ < 0.001
4718 reflections	Δρ_max_ = 0.17 e Å^−^^3^
308 parameters	Δρ_min_ = −0.16 e Å^−^^3^
0 restraints	Extinction correction: *SHELXL97* (Sheldrick, 2008), Fc^*^=kFc[1+0.001xFc^2^λ^3^/sin(2θ)]^-1/4^
Primary atom site location: structure-invariant direct methods	Extinction coefficient: 0.0091 (15)

### Special details

**Table:**

Geometry. All e.s.d.'s (except the e.s.d. in the dihedral angle between two l.s. planes) are estimated using the full covariance matrix. The cell e.s.d.'s are taken into account individually in the estimation of e.s.d.'s in distances, angles and torsion angles; correlations between e.s.d.'s in cell parameters are only used when they are defined by crystal symmetry. An approximate (isotropic) treatment of cell e.s.d.'s is used for estimating e.s.d.'s involving l.s. planes.
Refinement. Refinement of *F*^2^ against ALL reflections. The weighted *R*-factor *wR* and goodness of fit *S* are based on *F*^2^, conventional *R*-factors *R* are based on *F*, with *F* set to zero for negative *F*^2^. The threshold expression of *F*^2^ > σ(*F*^2^) is used only for calculating *R*-factors(gt) *etc*. and is not relevant to the choice of reflections for refinement. *R*-factors based on *F*^2^ are statistically about twice as large as those based on *F*, and *R*- factors based on ALL data will be even larger.

### Fractional atomic coordinates and isotropic or equivalent isotropic displacement parameters (Å^2^)

**Table:**

	*x*	*y*	*z*	*U*_iso_*/*U*_eq_	
O1	−0.1506 (2)	0.6822 (2)	0.16861 (11)	0.0805 (7)	
N1	0.3133 (2)	0.4580 (2)	0.35291 (12)	0.0619 (7)	
C1	0.1571 (3)	0.5157 (3)	0.19907 (15)	0.0668 (9)	
H1A	0.2051	0.4617	0.1800	0.080*	
N2	0.3059 (3)	0.2833 (2)	0.44379 (13)	0.0758 (8)	
O2	0.6891 (2)	0.8783 (2)	0.30391 (14)	0.1012 (9)	
C2	0.0404 (3)	0.5583 (3)	0.16694 (15)	0.0685 (9)	
H2A	0.0116	0.5337	0.1266	0.082*	
O3	0.4449 (2)	0.15376 (19)	0.49261 (11)	0.0832 (7)	
C3	−0.0322 (3)	0.6364 (3)	0.19481 (14)	0.0589 (8)	
C4	0.0133 (3)	0.6732 (3)	0.25457 (15)	0.0667 (9)	
H4A	−0.0355	0.7260	0.2741	0.080*	
C5	0.1310 (3)	0.6317 (3)	0.28509 (14)	0.0638 (9)	
H5A	0.1622	0.6587	0.3247	0.077*	
C6	0.2032 (3)	0.5510 (2)	0.25791 (14)	0.0555 (8)	
C7	−0.2053 (4)	0.6408 (4)	0.10938 (18)	0.1126 (15)	
H7A	−0.2877	0.6788	0.0961	0.169*	
H7B	−0.2213	0.5614	0.1122	0.169*	
H7C	−0.1439	0.6542	0.0796	0.169*	
C8	0.3347 (3)	0.5063 (3)	0.29208 (15)	0.0627 (9)	
H8A	0.3674	0.4465	0.2666	0.075*	
C9	0.4351 (3)	0.6013 (3)	0.29770 (15)	0.0607 (8)	
C10	0.5010 (3)	0.6272 (3)	0.24752 (17)	0.0716 (9)	
H10A	0.4877	0.5815	0.2123	0.086*	
C11	0.5857 (3)	0.7177 (3)	0.24720 (19)	0.0786 (10)	
H11A	0.6291	0.7321	0.2125	0.094*	
C12	0.6056 (3)	0.7869 (3)	0.2986 (2)	0.0740 (10)	
C13	0.5425 (4)	0.7637 (3)	0.35055 (18)	0.0772 (10)	
H13A	0.5571	0.8091	0.3859	0.093*	
C14	0.4574 (3)	0.6722 (3)	0.34940 (16)	0.0710 (9)	
H14A	0.4139	0.6576	0.3841	0.085*	
C15	0.7257 (4)	0.9251 (3)	0.2473 (2)	0.1188 (17)	
H15A	0.7840	0.9881	0.2570	0.178*	
H15B	0.6470	0.9496	0.2213	0.178*	
H15C	0.7705	0.8689	0.2257	0.178*	
C16	0.2141 (3)	0.3666 (3)	0.34414 (16)	0.0738 (10)	
H16A	0.2478	0.3063	0.3201	0.089*	
H16B	0.1326	0.3949	0.3208	0.089*	
C17	0.4359 (3)	0.4114 (3)	0.38666 (16)	0.0709 (10)	
H17A	0.5041	0.4693	0.3919	0.085*	
H17B	0.4680	0.3502	0.3628	0.085*	
C18	0.4094 (3)	0.3686 (3)	0.45001 (17)	0.0802 (10)	
H18A	0.4906	0.3370	0.4718	0.096*	
H18B	0.3822	0.4309	0.4746	0.096*	
C19	0.1841 (3)	0.3211 (3)	0.40618 (18)	0.0801 (11)	
H19A	0.1422	0.3794	0.4285	0.096*	
H19B	0.1223	0.2585	0.3992	0.096*	
C20	0.3342 (3)	0.1781 (3)	0.46539 (15)	0.0644 (9)	
C21	0.2268 (3)	0.0911 (3)	0.45499 (14)	0.0664 (9)	
H21A	0.1387	0.1139	0.4451	0.080*	
C22	0.2550 (3)	−0.0172 (3)	0.45954 (13)	0.0627 (9)	
H22A	0.3441	−0.0361	0.4703	0.075*	
C23	0.1603 (3)	−0.1113 (3)	0.44938 (13)	0.0598 (8)	
C24	0.2069 (4)	−0.2212 (3)	0.45818 (15)	0.0700 (9)	
H24A	0.2973	−0.2338	0.4698	0.084*	
C25	0.1216 (4)	−0.3106 (3)	0.44992 (16)	0.0783 (10)	
H25A	0.1554	−0.3829	0.4571	0.094*	
C26	−0.0126 (4)	−0.2975 (3)	0.43129 (16)	0.0758 (10)	
C27	−0.0588 (4)	−0.1881 (4)	0.42178 (19)	0.0875 (11)	
H27A	−0.1489	−0.1758	0.4090	0.105*	
C28	0.0254 (4)	−0.0979 (3)	0.43089 (18)	0.0828 (11)	
H28A	−0.0090	−0.0256	0.4245	0.099*	
C29	−0.1073 (4)	−0.3960 (3)	0.4196 (2)	0.1091 (14)	
H29A	−0.0593	−0.4651	0.4284	0.164*	
H29B	−0.1463	−0.3956	0.3767	0.164*	
H29C	−0.1763	−0.3898	0.4462	0.164*	

### Atomic displacement parameters (Å^2^)

**Table:**

	*U*^11^	*U*^22^	*U*^33^	*U*^12^	*U*^13^	*U*^23^
O1	0.0657 (15)	0.1064 (19)	0.0666 (15)	0.0123 (13)	−0.0028 (12)	0.0018 (13)
N1	0.0574 (15)	0.0505 (15)	0.0751 (19)	−0.0007 (13)	−0.0018 (13)	0.0095 (13)
C1	0.064 (2)	0.071 (2)	0.066 (2)	0.0023 (17)	0.0119 (17)	−0.0140 (17)
N2	0.0694 (18)	0.0615 (19)	0.091 (2)	−0.0016 (15)	−0.0121 (15)	0.0209 (15)
O2	0.0820 (18)	0.0766 (18)	0.142 (3)	−0.0202 (15)	0.0005 (16)	0.0127 (17)
C2	0.070 (2)	0.081 (2)	0.054 (2)	−0.0048 (19)	0.0091 (17)	−0.0109 (18)
O3	0.0748 (16)	0.0746 (16)	0.0935 (18)	0.0067 (13)	−0.0159 (14)	0.0169 (13)
C3	0.0573 (19)	0.069 (2)	0.0500 (19)	−0.0021 (17)	0.0072 (15)	0.0069 (16)
C4	0.069 (2)	0.075 (2)	0.056 (2)	0.0135 (18)	0.0081 (17)	−0.0032 (17)
C5	0.072 (2)	0.068 (2)	0.0492 (18)	0.0056 (18)	0.0014 (16)	−0.0064 (16)
C6	0.0589 (19)	0.0534 (18)	0.0545 (19)	−0.0033 (15)	0.0083 (15)	−0.0008 (15)
C7	0.082 (3)	0.178 (5)	0.071 (3)	0.013 (3)	−0.019 (2)	−0.003 (3)
C8	0.062 (2)	0.0521 (18)	0.074 (2)	0.0035 (16)	0.0099 (17)	−0.0032 (16)
C9	0.0533 (18)	0.058 (2)	0.071 (2)	0.0066 (16)	0.0072 (16)	0.0023 (17)
C10	0.062 (2)	0.070 (2)	0.086 (2)	0.0013 (18)	0.0222 (19)	−0.0059 (19)
C11	0.063 (2)	0.074 (2)	0.103 (3)	0.003 (2)	0.028 (2)	0.006 (2)
C12	0.052 (2)	0.059 (2)	0.110 (3)	−0.0011 (17)	0.005 (2)	0.016 (2)
C13	0.085 (2)	0.060 (2)	0.083 (3)	−0.004 (2)	−0.004 (2)	−0.0008 (19)
C14	0.075 (2)	0.065 (2)	0.073 (2)	−0.0015 (19)	0.0083 (18)	0.0036 (18)
C15	0.107 (3)	0.074 (3)	0.185 (5)	−0.013 (2)	0.058 (3)	0.019 (3)
C16	0.063 (2)	0.060 (2)	0.094 (3)	−0.0029 (17)	−0.0091 (18)	0.0113 (19)
C17	0.058 (2)	0.058 (2)	0.093 (3)	−0.0010 (16)	−0.0062 (18)	0.0048 (18)
C18	0.080 (2)	0.067 (2)	0.087 (3)	−0.0058 (19)	−0.0167 (19)	0.011 (2)
C19	0.066 (2)	0.068 (2)	0.104 (3)	0.0004 (18)	−0.002 (2)	0.029 (2)
C20	0.071 (2)	0.061 (2)	0.061 (2)	0.0073 (19)	0.0064 (18)	0.0068 (16)
C21	0.066 (2)	0.063 (2)	0.071 (2)	0.0108 (18)	0.0084 (17)	0.0160 (17)
C22	0.071 (2)	0.061 (2)	0.056 (2)	0.0088 (18)	0.0078 (16)	0.0108 (16)
C23	0.068 (2)	0.061 (2)	0.0508 (18)	0.0069 (18)	0.0082 (15)	0.0097 (15)
C24	0.079 (2)	0.063 (2)	0.066 (2)	0.009 (2)	0.0013 (18)	−0.0006 (17)
C25	0.099 (3)	0.062 (2)	0.071 (2)	0.008 (2)	−0.004 (2)	−0.0009 (17)
C26	0.097 (3)	0.069 (2)	0.062 (2)	−0.012 (2)	0.012 (2)	−0.0043 (18)
C27	0.066 (2)	0.089 (3)	0.107 (3)	0.000 (2)	0.010 (2)	0.003 (2)
C28	0.074 (2)	0.063 (2)	0.110 (3)	0.008 (2)	0.005 (2)	0.017 (2)
C29	0.121 (3)	0.097 (3)	0.109 (3)	−0.023 (3)	0.014 (3)	−0.006 (3)

### Geometric parameters (Å, °)

**Table:**

O1—C3	1.372 (3)	C13—H13A	0.9300
O1—C7	1.416 (4)	C14—H14A	0.9300
N1—C17	1.466 (4)	C15—H15A	0.9600
N1—C8	1.472 (4)	C15—H15B	0.9600
N1—C16	1.474 (4)	C15—H15C	0.9600
C1—C6	1.363 (4)	C16—C19	1.508 (4)
C1—C2	1.388 (4)	C16—H16A	0.9700
C1—H1A	0.9300	C16—H16B	0.9700
N2—C20	1.351 (4)	C17—C18	1.513 (4)
N2—C18	1.450 (4)	C17—H17A	0.9700
N2—C19	1.460 (4)	C17—H17B	0.9700
O2—C12	1.371 (4)	C18—H18A	0.9700
O2—C15	1.431 (5)	C18—H18B	0.9700
C2—C3	1.367 (4)	C19—H19A	0.9700
C2—H2A	0.9300	C19—H19B	0.9700
O3—C20	1.232 (4)	C20—C21	1.495 (4)
C3—C4	1.385 (4)	C21—C22	1.318 (4)
C4—C5	1.377 (4)	C21—H21A	0.9300
C4—H4A	0.9300	C22—C23	1.470 (4)
C5—C6	1.379 (4)	C22—H22A	0.9300
C5—H5A	0.9300	C23—C28	1.382 (4)
C6—C8	1.533 (4)	C23—C24	1.392 (4)
C7—H7A	0.9600	C24—C25	1.365 (4)
C7—H7B	0.9600	C24—H24A	0.9300
C7—H7C	0.9600	C25—C26	1.375 (5)
C8—C9	1.513 (4)	C25—H25A	0.9300
C8—H8A	0.9800	C26—C27	1.387 (5)
C9—C10	1.374 (4)	C26—C29	1.512 (5)
C9—C14	1.393 (4)	C27—C28	1.366 (5)
C10—C11	1.375 (4)	C27—H27A	0.9300
C10—H10A	0.9300	C28—H28A	0.9300
C11—C12	1.374 (5)	C29—H29A	0.9600
C11—H11A	0.9300	C29—H29B	0.9600
C12—C13	1.385 (5)	C29—H29C	0.9600
C13—C14	1.383 (4)		
			
C3—O1—C7	117.2 (3)	H15A—C15—H15C	109.5
C17—N1—C8	112.4 (2)	H15B—C15—H15C	109.5
C17—N1—C16	107.9 (2)	N1—C16—C19	111.0 (3)
C8—N1—C16	110.2 (2)	N1—C16—H16A	109.4
C6—C1—C2	121.6 (3)	C19—C16—H16A	109.4
C6—C1—H1A	119.2	N1—C16—H16B	109.4
C2—C1—H1A	119.2	C19—C16—H16B	109.4
C20—N2—C18	119.6 (3)	H16A—C16—H16B	108.0
C20—N2—C19	127.2 (3)	N1—C17—C18	109.9 (3)
C18—N2—C19	112.7 (3)	N1—C17—H17A	109.7
C12—O2—C15	117.2 (3)	C18—C17—H17A	109.7
C3—C2—C1	119.8 (3)	N1—C17—H17B	109.7
C3—C2—H2A	120.1	C18—C17—H17B	109.7
C1—C2—H2A	120.1	H17A—C17—H17B	108.2
C2—C3—O1	125.2 (3)	N2—C18—C17	111.0 (3)
C2—C3—C4	119.3 (3)	N2—C18—H18A	109.4
O1—C3—C4	115.5 (3)	C17—C18—H18A	109.4
C5—C4—C3	120.0 (3)	N2—C18—H18B	109.4
C5—C4—H4A	120.0	C17—C18—H18B	109.4
C3—C4—H4A	120.0	H18A—C18—H18B	108.0
C4—C5—C6	121.1 (3)	N2—C19—C16	110.8 (3)
C4—C5—H5A	119.4	N2—C19—H19A	109.5
C6—C5—H5A	119.4	C16—C19—H19A	109.5
C1—C6—C5	118.1 (3)	N2—C19—H19B	109.5
C1—C6—C8	121.2 (3)	C16—C19—H19B	109.5
C5—C6—C8	120.6 (3)	H19A—C19—H19B	108.1
O1—C7—H7A	109.5	O3—C20—N2	121.5 (3)
O1—C7—H7B	109.5	O3—C20—C21	120.7 (3)
H7A—C7—H7B	109.5	N2—C20—C21	117.8 (3)
O1—C7—H7C	109.5	C22—C21—C20	121.0 (3)
H7A—C7—H7C	109.5	C22—C21—H21A	119.5
H7B—C7—H7C	109.5	C20—C21—H21A	119.5
N1—C8—C9	113.0 (2)	C21—C22—C23	126.8 (3)
N1—C8—C6	110.4 (2)	C21—C22—H22A	116.6
C9—C8—C6	108.3 (2)	C23—C22—H22A	116.6
N1—C8—H8A	108.3	C28—C23—C24	116.9 (3)
C9—C8—H8A	108.3	C28—C23—C22	123.9 (3)
C6—C8—H8A	108.3	C24—C23—C22	119.2 (3)
C10—C9—C14	116.7 (3)	C25—C24—C23	120.8 (3)
C10—C9—C8	119.3 (3)	C25—C24—H24A	119.6
C14—C9—C8	123.7 (3)	C23—C24—H24A	119.6
C9—C10—C11	122.8 (3)	C24—C25—C26	122.4 (3)
C9—C10—H10A	118.6	C24—C25—H25A	118.8
C11—C10—H10A	118.6	C26—C25—H25A	118.8
C12—C11—C10	119.5 (3)	C25—C26—C27	116.8 (3)
C12—C11—H11A	120.3	C25—C26—C29	122.9 (4)
C10—C11—H11A	120.3	C27—C26—C29	120.2 (4)
O2—C12—C11	124.6 (4)	C28—C27—C26	121.3 (3)
O2—C12—C13	115.5 (4)	C28—C27—H27A	119.4
C11—C12—C13	119.9 (3)	C26—C27—H27A	119.4
C14—C13—C12	119.3 (3)	C27—C28—C23	121.8 (3)
C14—C13—H13A	120.3	C27—C28—H28A	119.1
C12—C13—H13A	120.3	C23—C28—H28A	119.1
C13—C14—C9	121.8 (3)	C26—C29—H29A	109.5
C13—C14—H14A	119.1	C26—C29—H29B	109.5
C9—C14—H14A	119.1	H29A—C29—H29B	109.5
O2—C15—H15A	109.5	C26—C29—H29C	109.5
O2—C15—H15B	109.5	H29A—C29—H29C	109.5
H15A—C15—H15B	109.5	H29B—C29—H29C	109.5
O2—C15—H15C	109.5		
			
C6—C1—C2—C3	−1.0 (5)	C12—C13—C14—C9	1.2 (5)
C1—C2—C3—O1	−178.1 (3)	C10—C9—C14—C13	−0.5 (5)
C1—C2—C3—C4	1.0 (5)	C8—C9—C14—C13	−174.8 (3)
C7—O1—C3—C2	3.5 (5)	C17—N1—C16—C19	60.7 (3)
C7—O1—C3—C4	−175.6 (3)	C8—N1—C16—C19	−176.3 (2)
C2—C3—C4—C5	0.5 (5)	C8—N1—C17—C18	177.1 (3)
O1—C3—C4—C5	179.6 (3)	C16—N1—C17—C18	−61.3 (3)
C3—C4—C5—C6	−2.0 (5)	C20—N2—C18—C17	118.8 (3)
C2—C1—C6—C5	−0.5 (5)	C19—N2—C18—C17	−53.6 (4)
C2—C1—C6—C8	−177.9 (3)	N1—C17—C18—N2	58.3 (4)
C4—C5—C6—C1	2.0 (5)	C20—N2—C19—C16	−119.4 (4)
C4—C5—C6—C8	179.4 (3)	C18—N2—C19—C16	52.4 (4)
C17—N1—C8—C9	−60.0 (3)	N1—C16—C19—N2	−56.1 (4)
C16—N1—C8—C9	179.7 (2)	C18—N2—C20—O3	2.9 (5)
C17—N1—C8—C6	178.5 (2)	C19—N2—C20—O3	174.1 (3)
C16—N1—C8—C6	58.2 (3)	C18—N2—C20—C21	−176.8 (3)
C1—C6—C8—N1	−124.9 (3)	C19—N2—C20—C21	−5.6 (5)
C5—C6—C8—N1	57.7 (4)	O3—C20—C21—C22	−17.6 (5)
C1—C6—C8—C9	110.9 (3)	N2—C20—C21—C22	162.1 (3)
C5—C6—C8—C9	−66.4 (4)	C20—C21—C22—C23	−178.7 (3)
N1—C8—C9—C10	156.6 (3)	C21—C22—C23—C28	2.5 (5)
C6—C8—C9—C10	−80.7 (3)	C21—C22—C23—C24	−177.9 (3)
N1—C8—C9—C14	−29.2 (4)	C28—C23—C24—C25	−1.2 (5)
C6—C8—C9—C14	93.4 (4)	C22—C23—C24—C25	179.2 (3)
C14—C9—C10—C11	0.2 (5)	C23—C24—C25—C26	1.6 (5)
C8—C9—C10—C11	174.7 (3)	C24—C25—C26—C27	−0.7 (5)
C9—C10—C11—C12	−0.6 (5)	C24—C25—C26—C29	177.6 (3)
C15—O2—C12—C11	19.9 (5)	C25—C26—C27—C28	−0.3 (5)
C15—O2—C12—C13	−162.4 (3)	C29—C26—C27—C28	−178.7 (4)
C10—C11—C12—O2	178.9 (3)	C26—C27—C28—C23	0.6 (6)
C10—C11—C12—C13	1.3 (5)	C24—C23—C28—C27	0.2 (5)
O2—C12—C13—C14	−179.4 (3)	C22—C23—C28—C27	179.7 (3)
C11—C12—C13—C14	−1.5 (5)		

### Hydrogen-bond geometry (Å, °)

**Table:**

*D*—H···*A*	*D*—H	H···*A*	*D*···*A*	*D*—H···*A*
C11—H11A···O1^i^	0.93	2.60	3.360 (4)	140
C22—H22A···O3^ii^	0.93	2.59	3.483 (4)	160

Symmetry codes: (i) *x*+1, *y*, *z*; (ii) −*x*+1, −*y*, −*z*+1.
